# Induction of specific adaptive immune responses by immunization with newly designed artificial glycosphingolipids

**DOI:** 10.1038/s41598-019-55088-9

**Published:** 2019-12-11

**Authors:** Tetsuya Okuda, Kayoko Shimizu, Satoshi Hasaba, Mutsuhiro Date

**Affiliations:** 1 Bio-Design Research Group, Bioproduction Research Institute, National Institute of Advanced Industrial Science and Technology (AIST), Central 6, 1-1-1 Higashi, Tsukuba, Japan; 2 Diagnostics Research Laboratories, Diagnostics Technical Service & Research Operations, Diagnostics Division, FUJIFILM Wako Pure Chemical Corporation, 6-1 Takada, Amagasaki, Hyogo Japan

**Keywords:** Antibody generation, Conjugate vaccines

## Abstract

We previously found that artificial glycosphingolipids (artGSLs) containing very-long-chain fatty acids behave as strong immunogens in mice and promote the production of antibodies recognizing the oligosaccharide portion of artGSLs as the epitope. Here, we report that the oligosaccharide structure of artGSLs influences these immunogenic properties. We evaluated the antibody-inducing activity of artGSLs with different oligosaccharide structures in mice and found strong IgG-inducing activity only with an artGSL containing a core-fucosylated tetraoligosaccharide (Manβ1,4GlcNAcβ1,4[Fucα1,6]GlcNAc). To characterize the immunogenic properties of this artGSL, we analyzed various derivatives and found that the non-reducing terminal mannose structure was critical for the antibody-inducing activity. These artGSLs also exhibited IgG-inducing activity dependent on co-administration of lipid A adjuvant, but no cytokine-inducing activity similar to α-galactosylceramide was detected. Furthermore, repetitive immunization with the artGSL promoted the production of antibodies against a core-fucosylated α-fetoprotein isoform (AFP-L3) known as a hepatocellular carcinoma–specific antigen. These results indicate that the newly designed artGSLs specifically induce adaptive immune responses and promote antibody production by B cells, which can be utilized to develop anti-glycoconjugate antibodies and cancer vaccines targeting tumor-associated carbohydrate antigens.

## Introduction

Of the glycoconjugates synthesized in mammalian cells, glycosphingolipids (GSLs) are known to exhibit immunogenicity in immunized animals^1^. This activity was identified by analyzing anti-glycoconjugate monoclonal antibodies isolated from animals immunized with cancer cells^1–3^, which revealed that intracellular GSLs function as efficient immunogens for inducing antibody production. Recent studies also revealed that α-linked monosaccharyl ceramides such as α-galactosylceramide (αGalCer) isolated from marine sponges and bacteria activate mammalian natural killer T (NKT) cells and promote cytokine production^4–7^. In addition, CD1d expressed by antigen-presenting cells (APCs) was identified as the receptor for these NKT cell ligand GSLs^5,7^. Rodent GSLs with an α-galactosyl structure that exhibit this activity^7–9^ are considered natural ligands necessary for the development of NKT cells. Although the role of GSLs in innate immunity has been extensively investigated, the antibody-inducing activity of mammalian GSLs has received relatively little research attention.

We previously demonstrated that stimulation of human vascular endothelial cells with tumor necrosis factor-α promotes GSL synthesis and that the synthesized GSLs, particularly globotetraosylceramide (globoside/Gb4Cer), predominantly contain C24 very-long-chain fatty acids in the ceramide portion^10,11^. Surprisingly, we found that C24-Gb4Cer exhibits potent antibody-inducing activity in mice, including efficient induction of anti-Gb4Cer IgG synthesis^12,13^. Using immunized mice, we obtained monoclonal IgM and IgG3 antibodies that specifically recognize Gb4Cer with high affinity^12–14^. Further modeling experiments using newly designed artificial glycosphingolipids (artGSLs) composed of simple ceramide mimetics and 6′-sialyl LacNAc revealed that the C24 fatty acid structure is associated with the high antibody-inducing activity of the artGSLs^1,12^. Mice immunized with the artGSLs efficiently produced antibodies recognizing glycoproteins containing 6′-sialyl LacNAc^12^.

**Figure 1:**
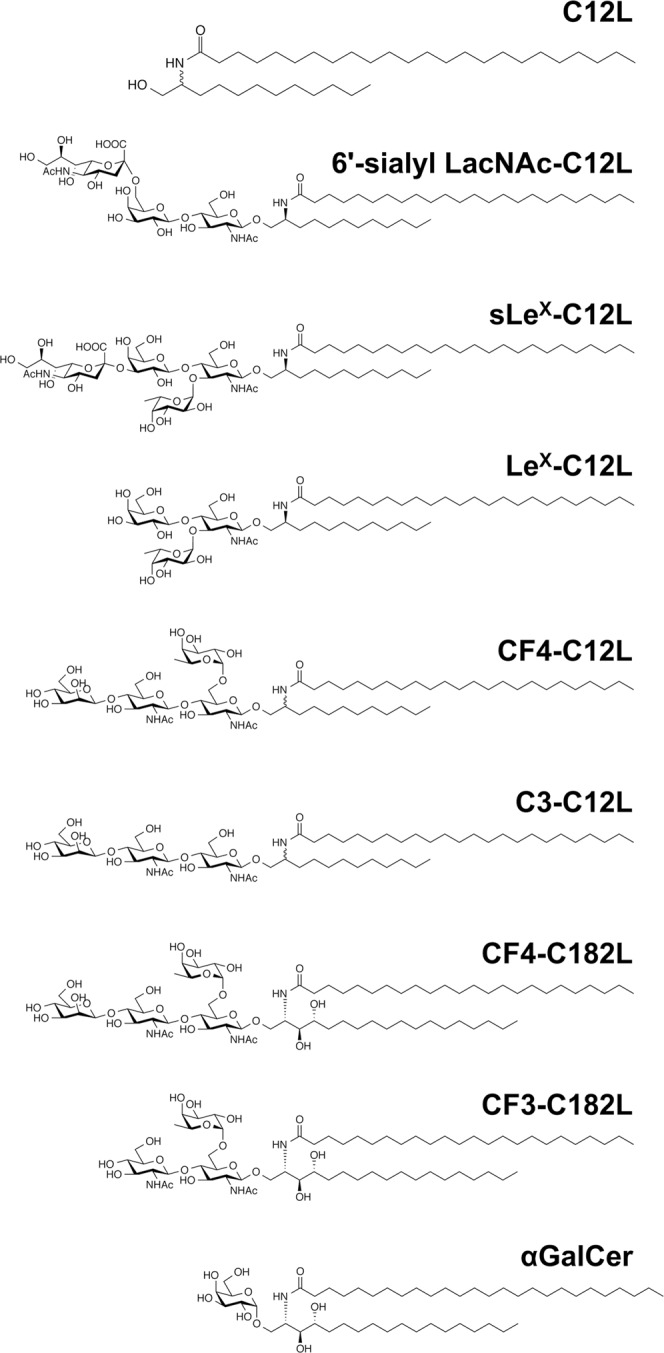
Chemical structures of artGSLs. The structures of C12L, artGSLs, and αGalCer used in this study are shown. The ceramide mimetic C12L is composed of a saturated C12-sphingosine mimetic and lignoceric acid (C24:0). The ceramide mimetic C182L is composed of C18-phytosphingosine and lignoceric acid. These ceramide mimetics are bound to the oligosaccharide via a β-linkage. The ceramide in αGalCer is composed of C18-phytosphingosine and hexacosanoic acid (C26:0) and bound to galactose via an α-linkage. Abbreviations: 6′-sialyl LacNAc, Neu5Acα2,6Galβ1,4GlcNAc; sLe^X^, sialyl Lewis^X^/Neu5Acα2,3Galβ1,4(Fucα1,3)GlcNAc; Le^X^, Lewis^X^/Galβ1,4(Fucα1,3)GlcNAc; CF4, core-fucosylated tetrasaccharide/Manβ1,4GlcNAcβ1,4(Fucα1,6)GlcNAc.

Of the ceramide mimetics we generated, C12L exhibited the highest immunogenicity (Fig. 1). In this study, we synthesized several artGSLs composed of C12L and various oligosaccharides and analyzed the effect of the oligosaccharide portion on antibody-inducing activity. The effect of altering the sphingosine portion was also analyzed. We found that artGSLs containing a core-fucosylated tetrasaccharide (CF4: Manβ1,4GlcNAcβ1,4[Fucα1,6]GlcNAc) exhibited potent IgG-inducing activity that was dependent on the non-reducing terminal mannose structure and co-administration of lipid A adjuvant. Unlike αGalCer, these artGSLs did not induce cytokine production in mice. Furthermore, repetitive immunization with CF4-C12L induced the production of antibodies against a core-fucosylated α-fetoprotein isoform (AFP-L3) known to be a hepatocellular carcinoma–specific antigen^15^. Collectively, these results indicate that the newly designed artGSLs specifically induce adaptive immune responses and promote antibody production by B cells, which can be exploited to develop anti-glycoconjugate antibodies and cancer vaccines targeting tumor-associated carbohydrate antigens.

## Results

### Design of new artGSLs containing very-long-chain fatty acids

We previously analyzed the antibody-inducing activity artGSLs composed of 6′-sialyl LacNAc (Neu5Acα2,6Galβ1,4GlcNAc) and C12L (Fig. 1) or C12L analogues and found that the very-long-chain fatty acid structure of C12L is essential for the induction of potent antibody responses by artGSLs^1,12^. In this study, we analyzed the effect of the oligosaccharide structure of various C12L conjugates on antibody-inducing activity in comparison with 6′-sialyl LacNAc-C12L and several oligosaccharide conjugates of C12L. From a practical perspective, oligosaccharides containing a sialyl Lewis^X^ (sLe^X^), Lewis^X^ (Le^X^), or core fucose epitope known to be carcinoembryonic antigens were selected (Fig. 1). sLe^X^ is expressed on lymphocytes and in gastrointestinal tumors^16–18^ and exhibits adhesive properties that play a role in the metastasis of these cancers. Le^X^, also known as SSEA-1, is a precursor of sLe^X^ and a well-characterized cell surface marker of mouse embryo and stem cells^19^. Core fucose forms the core structure of *N*-linked oligosaccharides in mammalian glycoproteins such as α-fetoprotein isoform AFP-L3, a hepatocellular carcinoma–specific antigen^15^. The core fucose structure of immunoglobulins has also been associated with anti-cancer activity^20,21^. To further analyze core fucose, we designed and prepared a C12L conjugate of core-fucosylated tetrasaccharide (CF4-C12L) and a non-fucosylated analogue (C3-C12L) as a negative control. For further analysis of CF4-C12L, we also prepared a phytosphingosine analogue (CF4-C182L) and trisaccharide analogue (CF3-C182L). As tri or tetrasaccharide length target oligosaccharide was enough for induction of antibodies that recognize the target oligosaccharide with high specificity^12–14^, we designed artGSLs containing tri or tetrasaccharides in this study.

### Antibody-inducing activity of artGSLs containing various oligosaccharides

**Figure 2:**
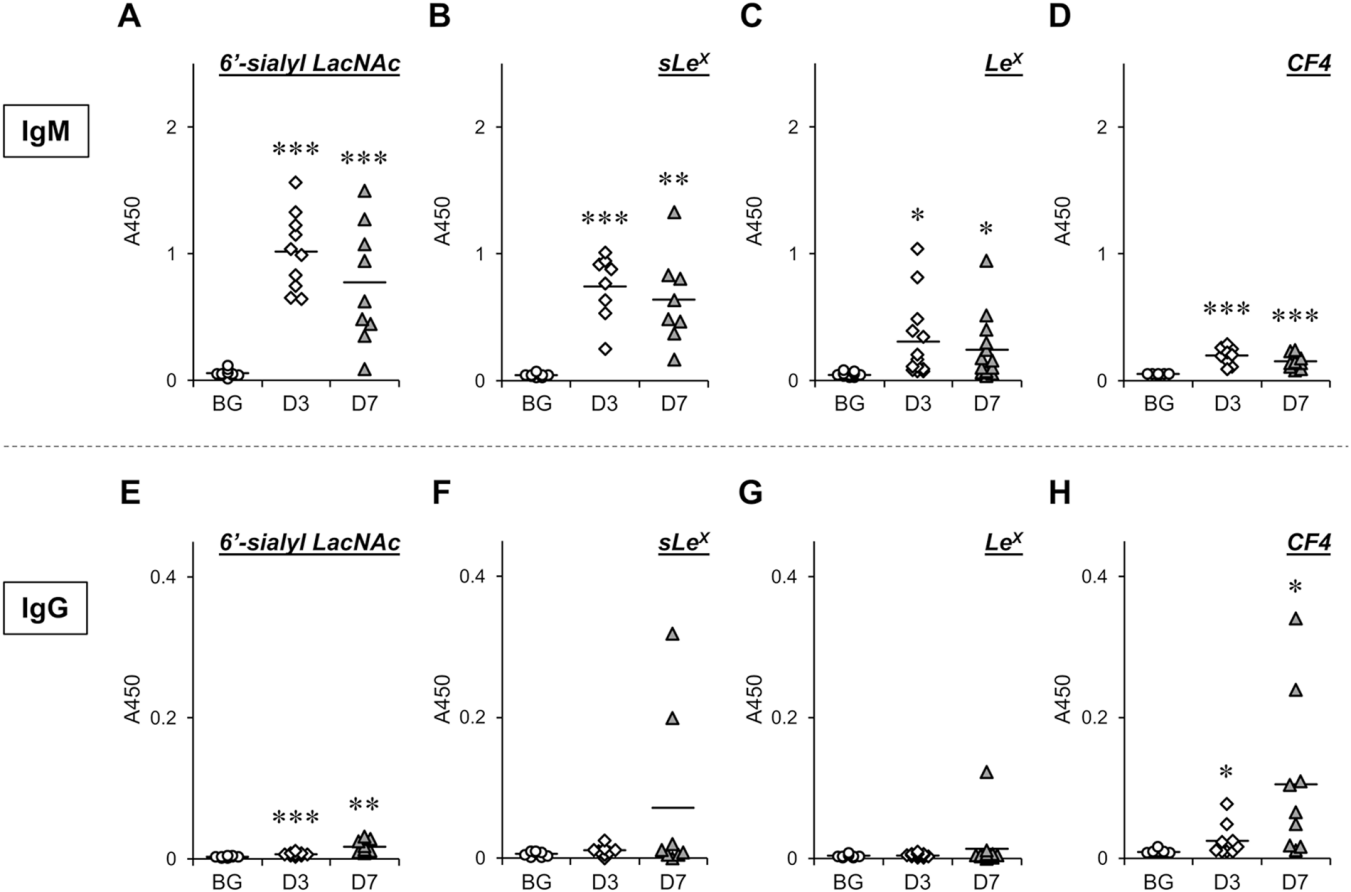
Titers of immunogen-specific serum IgM and IgG in artGSL-immunized mice. Mice were immunized with 6′-sialyl LacNAc-C12L (**A,E**), sLe^X^-C12L (**B,F**), Le^X^-C12L (**C,G**), or CF4-C12L (**D,H**), and serum was prepared 3 days (D3) or 7 days (D7) after the final immunization. BG, serum from non-treated mice. Titers of serum IgM (**A**–**D**) and IgG (**E**–**H**) against artGSLs used for immunization were determined by ELISA. Average absorbance values are indicated as horizontal bars. *P < 0.05, **P < 0.01, **P < 0.001 BG vs. D3 or D7 (n = 8–13).

**Figure 3:**
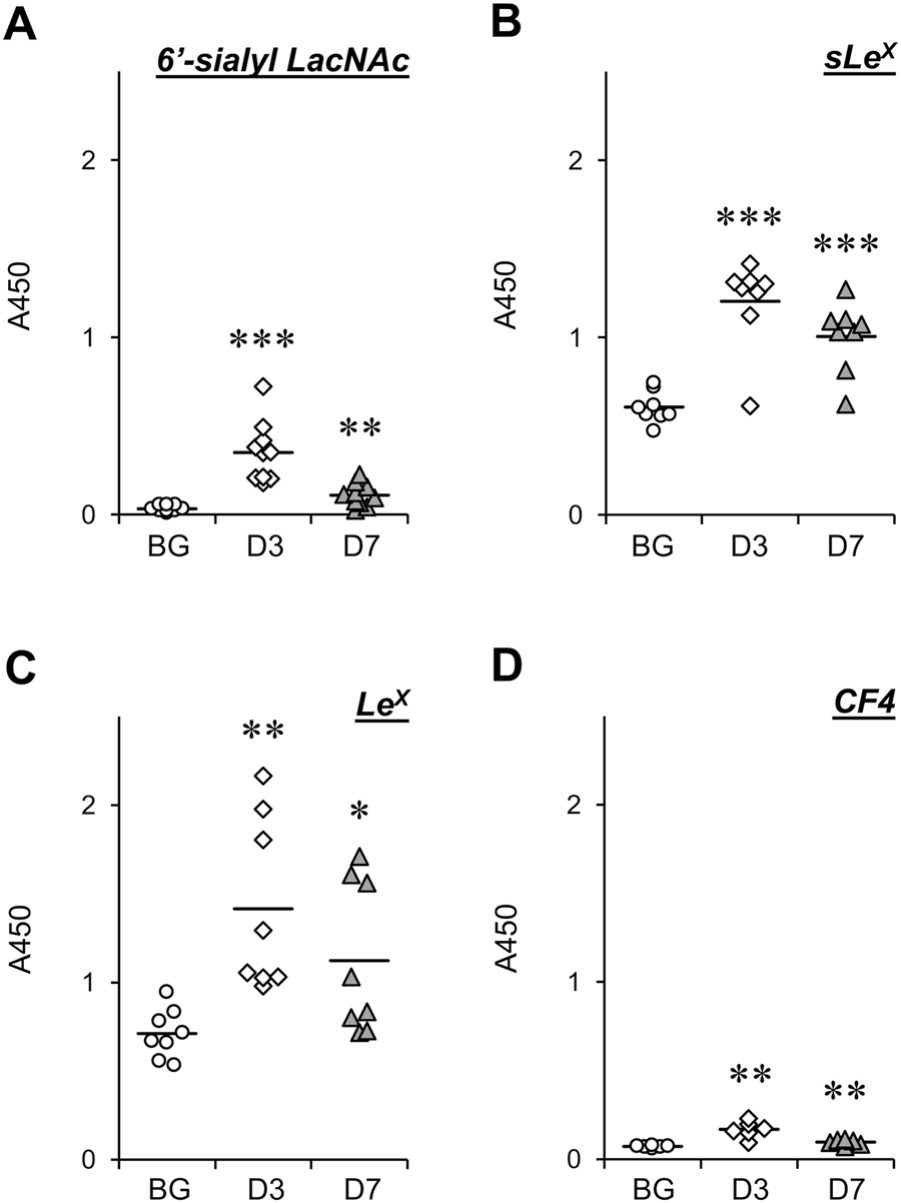
Titers of serum glycoprotein-specific IgM in artGSL-immunized mice. Mice were immunized with 6′-sialyl LacNAc-C12L (**A**), sLe^X^-C12L (**B**), Le^X^-C12L (**C**), or CF4-C12L (**D**), and serum was prepared 3 days (D3) or 7 days (D7) after the final immunization. BG, serum from non-treated mice. Titers of serum IgM against glycoproteins containing oligosaccharides of identical structure as artGSLs used for immunization were determined by ELISA. Glycoproteins containing 6′-sialyl LacNAc (fetuin [**A**]), sLe^X^ and Le^X^ (glycoproteins extracted from HL-60 cells [**B,C**]), and CF4 (thyroglobulin [**D**]) were used as immobilized antigens for ELISA, respectively. Average absorbance values are indicated as horizontal bars. *P < 0.05, **P < 0.01, **P < 0.001 BG vs. D3 or D7 (n = 8–10).

Mice were immunized with C12L conjugates containing 6′-sialyl LacNAc, sLe^X^, Le^X^, and CF4 using a liposome immunization method established by Brodin *et al*.^22^. The second booster immunization was performed intraperitoneally 2 weeks after the first subcutaneous immunization, and the antibody titer in the serum was evaluated by enzyme-linked immunosorbent assay (ELISA) on days 3 and 7 after the second immunization (Figs. 2, 3). Although our previous study using 6′-sialyl LacNAc-C12L showed that immunization of mice with this artGSL immediately induced the production of serum IgM and IgG against 6′-sialyl LacNAc as the epitope, the serum IgG titer was markedly lower than that of IgM^12^. In the present study, we found that the ratio of IgG to IgM depended on the oligosaccharide structure of the artGSLs. Figure 2A–D shows that 6′-sialyl LacNAc-C12L, sLe^X^-C12L, and Le^X^-C12L strongly induced the generation of IgM against these respective immunogens. While serum IgG titers appeared rapidly after the second immunization (Fig. 2E–H), these titers were relatively low (6′-sialyl LacNA-C12L) or exhibited large individual differences between mice (sLe^X^-C12L and Le^X^-C12L). However, CF4-C12L strongly induced the generation of serum IgG against the immunogen in almost all mice (Fig. 2H). The anti-immunogen IgG titers of serum from CF4-C12L–immunized mice significantly increased from 3 days after booster immunization, and the average value on the 7 days after was 6 times higher than that of 6′-sialyl LacNA-C12L. The IgG titer of 6 of 9 mice showed over 5 times higher than that of average level of backgrounds, such immunogenicity was not observed in other artGSLs. In contrast, IgM titers were relatively lower compared with the other artGSL-immunized mice (Fig. 2D).

**Figure 4:**
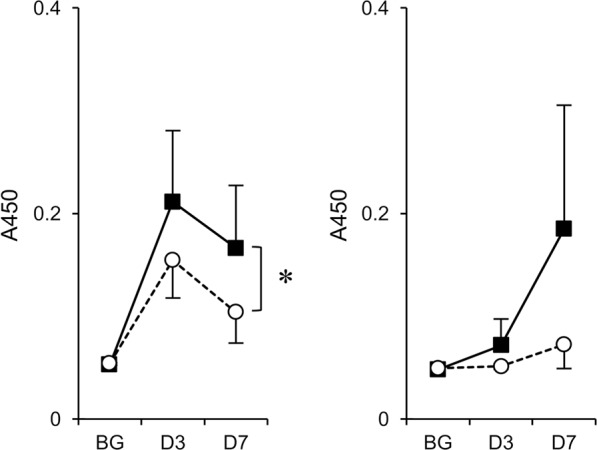
Comparison of serum titers of antibodies against CF4-C12L and C3-C12L in mice immunized with CF4-C12L. Mice were immunized with CF4-C12L, and serum was prepared 3 days (D3) or 7 days (D7) after the final immunization. BG, serum from non-treated mice. Titers of serum IgM (*left panel*) and IgG (*right panel*) against CF4-C12L (*closed squares with solid line*) and C3-C12L (*open circles with dotted line*) were determined by ELISA. Error bars: mean ± S.D. (n = 6). *P < 0.05 anti-CF4-C12L vs. anti-CF3-C12L.

Our previous study also showed that artGSL-induced antibodies react with glycoproteins with oligosaccharide structures identical to those of the artGSLs used for immunization. Thus, we also analyzed the titers of serum antibodies against glycoproteins containing 6′-sialyl LacNAc (fetuin), sLe^X^ and Le^X^ (glycoproteins extracted from HL-60 cells), and CF4 (thyroglobulin)^16,23,24^ in artGSL-immunized mice using ELISAs (Fig. 3). Although no induction of IgG was detected under the experimental conditions used, significant induction of IgM against glycoproteins containing oligosaccharides identical to those of the immunogens was observed in all artGSL-immunized mice. Although significant induction of IgM against thyroglobulin was detected in mice immunized with CF4-C12L, the titers were lower than those of other artGSL-immunized mice (Fig. 3D). As only core fucose forms the root structure of *N*-linked oligosaccharides, while the other oligosaccharides are terminal structures, it was considered that the low IgM titers were related to structural issues. Thus, to confirm that the antibodies generated in CF4-C12L–immunized mice recognized core fucose as the epitope, we analyzed the reactivity of the antibodies to the precursor, C3-C12L, which does not contain core fucose (Fig. 4). The reactivity of IgM generated in response to CF4-C12L was significantly higher than that of the IgM generated in response to C3-C12L; a similar tendency was observed with IgG.

These results demonstrated that conjugation of C12L efficiently enhances the immunogenicity of various oligosaccharides in mice and that conjugates/artGSLs are efficient immunogens for inducing the generation of antibodies against glycoproteins containing target oligosaccharides. Furthermore, we found that the oligosaccharide structure influences the ratio of IgG to IgM induction, and more specifically, class switching.

### Characterization of the immunogenic properties of CF4-C12L and its derivatives

**Figure 5:**
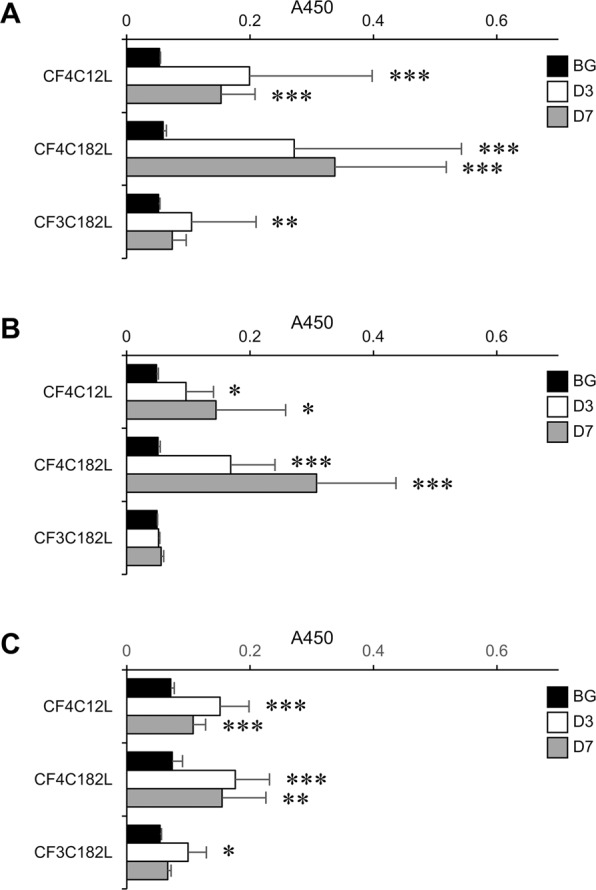
Serum antibody titers in CF4-C182L– and CF3-C182L–immunized mice. Mice were immunized with CF4-C182L or CF3-C182L, and serum was prepared 3 (*open bars*) or 7 (*gray bars*) days after the final immunization. *Closed bars*, serum from non-treated mice. Titers of serum IgM (**A**) and IgG (**B**) against each immunogen were determined by ELISA. (**C**) Titers of serum IgM against thyroglobulin. Titers of serum antibodies in CF4-C12L–immunized mice are shown for reference. Error bars: mean ± S.D. (n = 6–7). *P < 0.05, **P < 0.01, **P < 0.001 BG vs. D3 or D7.

To determine the key structure mediating the high IgG-inducing activity of CF4-C12L, we prepared various CF4-C12L derivatives (Fig. 1, CF4-C182L and CF3-C182L) and analyzed their immunogenic properties. As our previous analysis using 6′-sialyl LacNAc-C12L revealed that the C24:0 lignoceric acid of C12L was essential for IgG-inducing activity^12^, in the present study, we examined the effect of altering the sphingoid base or oligosaccharide structure of CF4-C12L. First, we examined the antibody-inducing activity of CF4-C182L, a phytosphingosine analogue of CF4-C12L, in comparison to CF4-C12L (Fig. 5). We previously examined how the length of the alkyl chain of the sphingosine mimetic portion of C12L affects the immunogenicity of 6′-sialyl LacNAc-C12L and found that the C12L conjugate exhibited higher immunogenicity than the short (8 carbon length) and long (16 carbon length) sphingosine analogues^12^. As the hydroxyl group of the phytosphingosine portion is known to play an important role in determining the immunogenicity of αGalCer^25–27^, in the present study, we examined the immunogenicity of the phytosphingosine analogue of C12L. In Fig. 5B, we observed a significant enhancement of serum IgG titers against immunogen in mice immunized with CF4-C182L compared to that in CF4-C12L–immunized mice (D3, *P* < 0.05; D7, *P* < 0.01; anti-CF4-C12L vs. anti-CF4-C182L). Further analysis of the endpoint titers in the serum from three mice with high IgG titer against CF4-conjugates (Fig. S1) also showed that the endopoint titer of CF4-C182L tended to be lower than that of CF4-C12L (CF4-C12L, from 1000-fold to 2000-fold; CF4-C182L, 2000-fold).

Next, we prepared an oligosaccharide analogue of CF4-C182L by removing the non-reducing terminal mannose (CF3-C182L) and analyzed its antibody-inducing activity in mice. Surprisingly, we found that removal of the terminal mannose resulted in a marked reduction in the antibody-inducing activity of CF3-C182L; in particular, the IgG titer was markedly reduced by removal of the terminal mannose (Fig. 5B). This result indicates that the terminal mannose plays a critical role in antibody induction, particularly the IgG-inducing activity of the CF4 conjugates.

**Figure 6:**
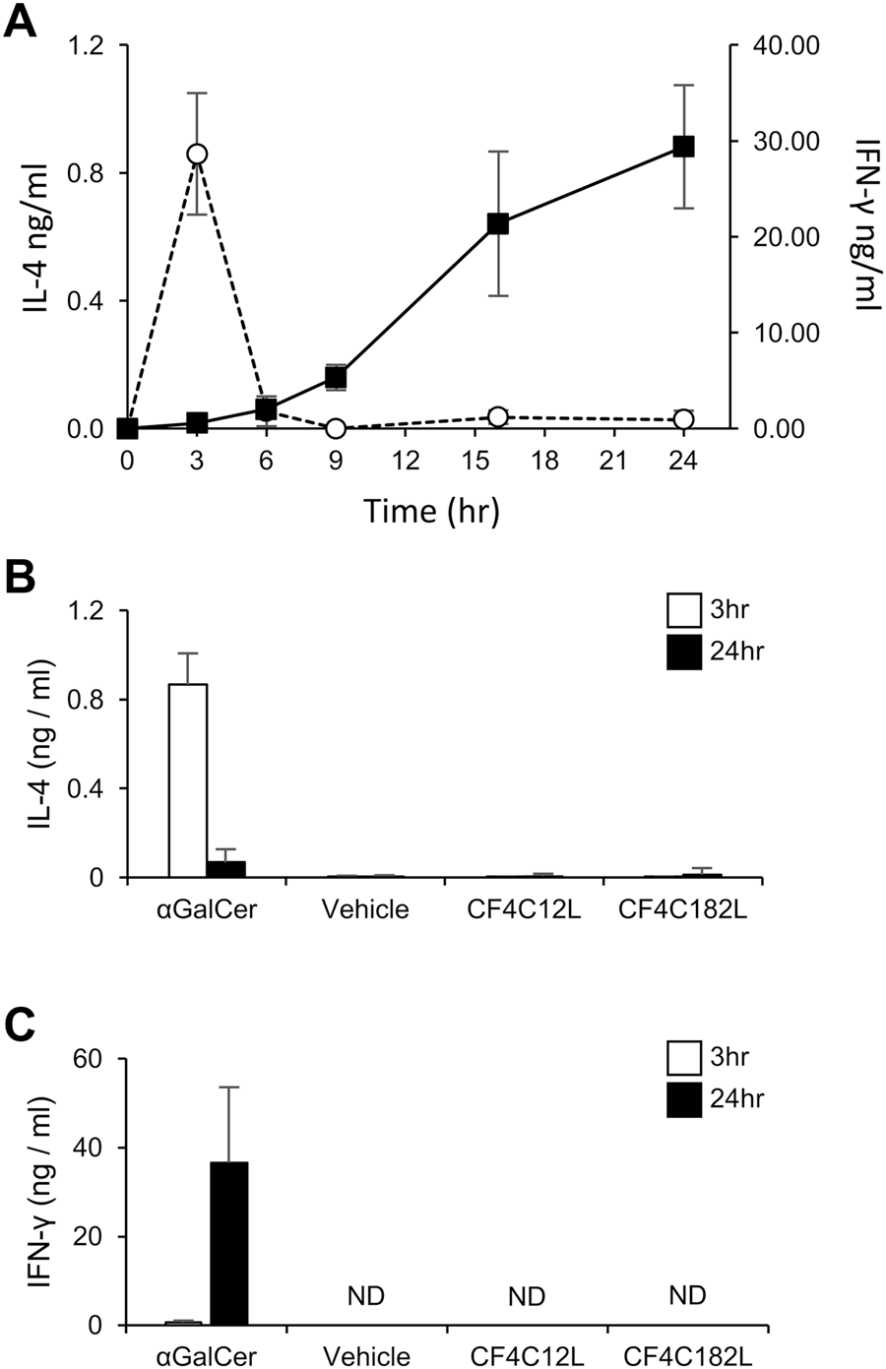
Comparison of cytokine-inducing activity of αGalCer and artGSLs. Time course of change in serum IL-4 (*open circles with dotted line*) and IFN-γ (*closed squares with solid line*) levels in mice administered αGalCer (**A**). Serum levels of IL-4 (**B**) and IFN-γ (**C**) at 3 (*open bars*) or 24 (*closed bars*) hours after administration of CF4-C12L and CF4-C182L. Vehicle indicates serum cytokine levels in mice not administered glycosphingolipid-containing liposomes. Error bars: mean ± S.D. (n = 5). ND, not detected.

As conjugates of CF4 were found to induce IgG with high efficiency, we hypothesized that the conjugates induce the production of cytokines related to class switching, similar to αGalCer^4,5,7^. Thus, we examined serum levels of IL-4 and IFN-γ, representative cytokines induced by αGalCer, in mice administered CF4 conjugates. In the C3H/HeN mice used in the immunization experiment, αGalCer administration transiently increased the IL-4 level at 3 hours and gradually increased the IFN-γ level between 9 and 24 hours post-administration (Fig. 6A). We therefore examined serum levels of IL-4 and IFN-γ at 3 and 24 hours after administration of the CF4 conjugates (Fig. 6B,C). In contrast to αGalCer, no production of these cytokines was observed in mice administered the CF4 conjugates. In addition, we also did not detect the production of antibodies against αGalCer in αGalCer-immunized mice (Fig. S2). These results indicate that the CF4 conjugates do not stimulate innate immunity, but they do induce an adaptive immune response.

**Figure 7:**
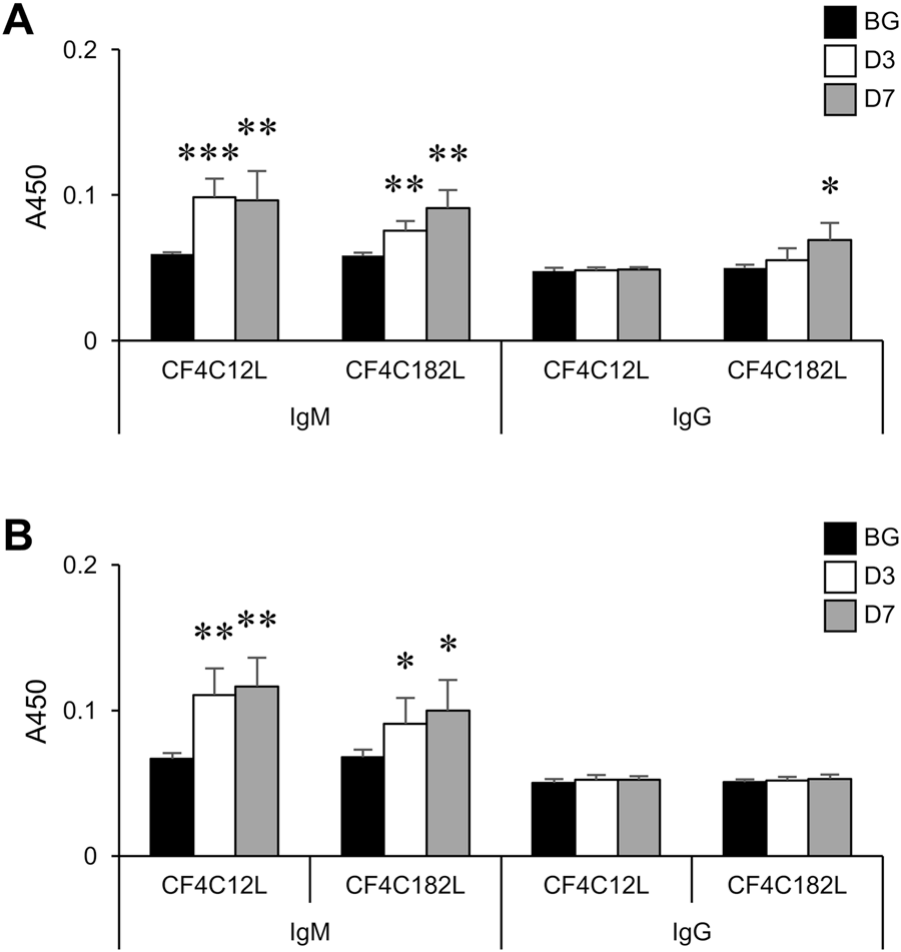
Effect of lipid A on antibody-inducing activity of CF4 conjugates. Mice were immunized with CF4-C12L or CF4-C182L without lipid A, and serum was prepared 3 (*open bars*) or 7 (*gray bars*) days after the final immunization. *Closed bars*, serum from non-treated mice. Titers of serum IgM and IgG against each immunogen (*upper panel*) or thyroglobulin (*lower panel*) were determined by ELISA. Error bars: mean ± S.D. (n = 6). *P < 0.05, **P < 0.01, ***P < 0.001 BG vs. D3 or D7.

To examine antibody induction, we simultaneously administered immunogens with lipid A as an adjuvant. In order to analyze the antibody-inducing activity of CF4 conjugates alone, mice were immunized with liposomes containing CF4 conjugates but without lipid A, and the serum antibodies titers were then determined. Although IgM-inducing activity remained in the absence of lipid A, IgG-inducing activity was almost abolished (Fig. 7), indicating that the IgG-inducing activity of the CF4 conjugates is dependent on co-immunization with lipid A.

### Induction of antibodies to cancer-specific glycoproteins by immunization with CF4-C12L

AFP-L3 is a core-fucosylated α-fetoprotein isoform and hepatocellular carcinoma–specific marker^15^. If immunization with CF4 conjugates could induce the generation of antibodies that specifically react with AFP-L3, it would be possible to produce monoclonal antibodies against AFP-L3 for diagnostic use, and this could also facilitate the development of vaccines to prevent this type of cancer.

**Figure 8:**
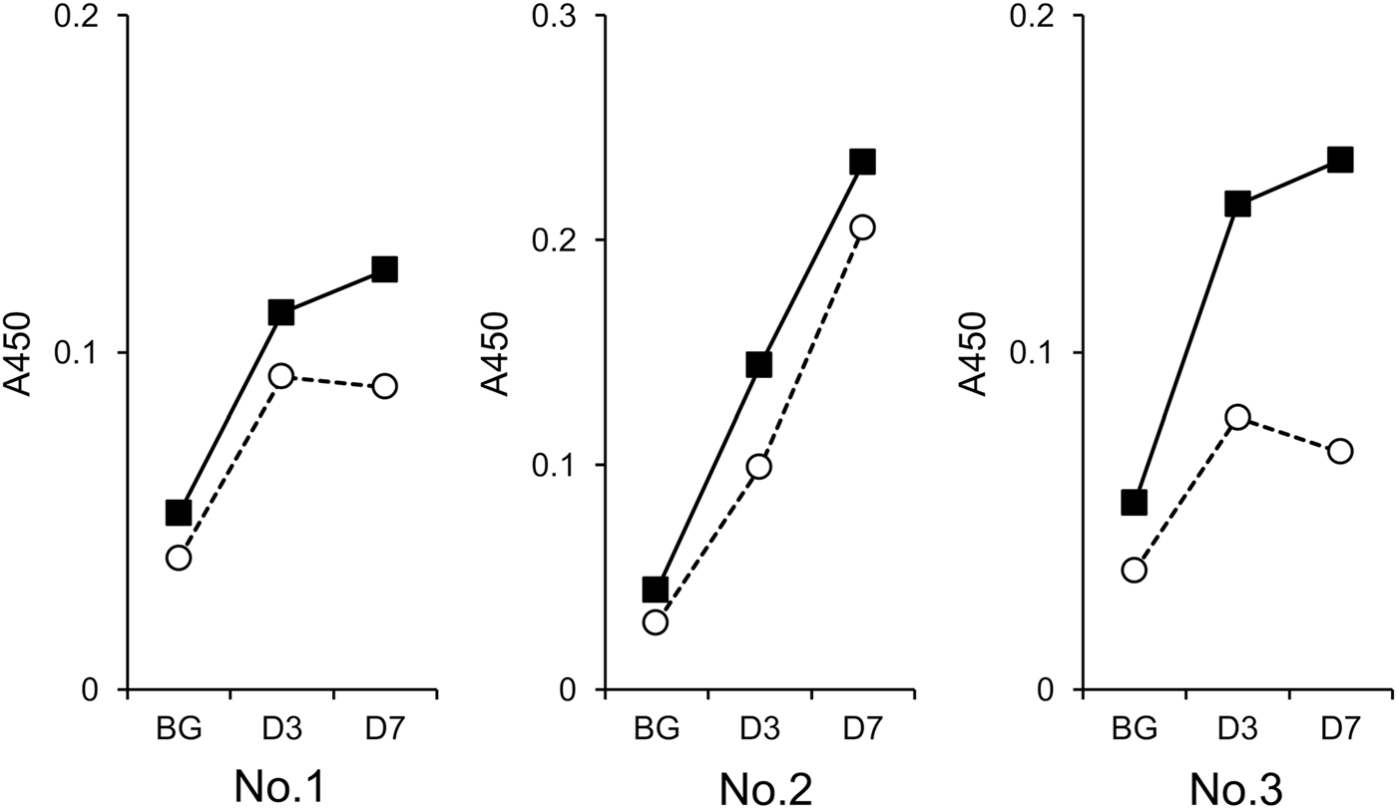
Titers of serum antibodies against AFP-L3 in CF4-C12L–immunized mice. Serum was prepared from mice immunized more than 4 times with CF4-C12L at 3 days (D3) or 7 days (D7) after the final immunization. BG, serum from non-treated mice. Total titer of serum immunoglobulin against AFP-L3 (*closed squares with solid line*) or AFP-L1 (*open circles with dotted line*) was determined by ELISA. Three mice (Nos. 1, 2, and 3) were analyzed.

To examine the generation of antibodies against AFP-L3, mice were repeatedly immunized with CF4-C12L, and titers of serum antibodies against AFP-L3 were analyzed by ELISA. As preliminary experiments showed that the antibody-inducing activity of CF4-C182L was not enhanced by repetitive immunization (Fig. S3), we used CF4-C12L for this experiment. We started this experiment using 6 mice, and finally analyzed 3 mice that showed strong antibody titers against immunogen. After repetitive immunization with CF4-C12L, antibodies against AFP-L3 were clearly detected in three mice, as measured by an increase in total immunoglobulin (Fig. 8). Particularly high reactivity to AFP-L3 relative to AFP-L1, the non-fucosylated α-fetoprotein isoform, was observed in one of the three mice (Fig. 8, No. 3). This result suggests that CF4-C12L could be an effective immunogen for practical use in the diagnosis and treatment of hepatocellular carcinoma. In further preliminary experiments using serum from mouse No. 3, IgM and IgG3 in which the κ light chain was detected were detected as the major serum immunoglobulins; other IgG subclasses were detected as minor components (Fig. S4). As the subclass of monoclonal antibodies obtained previously using a GSL with similar structure of CF4-conjugates was also IgG3^13^, these results suggest that the immune responses induced by the CF4-conjugates may be a T-independent response.

## Discussion

The results of this study demonstrate that altering the oligosaccharide structure of mammalian GSL analogues enhances their IgG-inducing activity. We previously found that both mammalian and artificial GSLs containing very-long-chain fatty acids induce the generation of IgG that reacts with these oligosaccharides as the epitope, but the IgG-inducing activity was relatively lower than the IgM-inducing activity^12,13^. In contrast, newly designed artGSL derivatives containing CF4 exhibited strong IgG-inducing activity, and the finding that certain structural features of the oligosaccharide portion of GSLs are associated with their IgG-inducing activity is novel. In previous studies of innate immunity using αGalCer analogues, elongation of the monosaccharides to oligosaccharides was thought to weaken the immunogenicity of the analogues^28^. However, our study demonstrates that certain oligosaccharides can induce a specific adaptive immune response, which overturns a conventional concept of mammalian immunology. This finding not only contributes to progress in immunology research but might also facilitate advances in drug development, such as the design of novel glycoconjugate immunogens and vaccines.

Of the artGSL oligosaccharide structures examined in this study, CF4 exhibited the highest IgG-inducing activity (Fig. 2). This activity was abolished by removal of the non-reducing terminal mannose (Fig. 5B), indicating that this structure is necessary for the IgG-inducing activity of artGSLs. IgG production by B cells involves class switching via APC-mediated interaction between B and T cells. As the mannose receptor, a C-type lectin present on the surface of APCs, recognizes the mannose structure of glycoconjugates^29^, APCs could recognize artGSLs containing mannose more efficiently. Furthermore, the very-long-chain fatty acid structure of αGalCer is known to play an important role in recognition involving CD1d, an antigen-presenting molecule expressed by APCs^7,30–32^. A previous model study also reported that very-long-chain alkyl structures of synthetic lipids are required for transport to lysosomes, where GSLs dock with CD1d^33^. These observations indicate that CF4-C12L and CF4-C182L possess several structural advantages that facilitate more-efficient recognition by APCs, resulting in strong induction of IgG responses.

Our results also indicate that GSLs containing very-long-chain fatty acids exhibit strong antibody-inducible activity in mice, where they can become self-antigens. Further elucidation of the physiologic functions of anti-GSL antibodies may resolve this issue. We previously reported that GSLs containing very-long-chain fatty acids derived from human vascular endothelial cells also exhibit potent antibody-inducing activity^12,13^, and the results of the present study demonstrate that the potent antibody-inducing activity of artGSLs containing very-long-chain fatty acid depends on the co-administration of lipid A, a component of lipopolysaccharide (LPS) (Fig. 7). In mice administered LPS, levels of GSLs containing very-long-chain fatty acids increase in vascular endothelial cells^10,11,34,35^, which is thought to result in the suppression of inflammation^35^. These results suggest the possibility that levels of GSLs containing very-long-chain fatty acids increase following infection with LPS-producing gram-negative bacteria, which in turn induces the production of IgM and IgG that exert anti-inflammatory effects in coordination with lipid A derived from LPS.

Tumor necrosis factor-α promotes the synthesis of GSLs containing very-long-chain fatty acids in human vascular endothelial cells through the transcriptional up-regulation of GSL synthesis–related genes^10,11^, including the ceramide synthase 2 gene. As mice deficient in this gene develop hepatocellular carcinoma^36^, ceramide synthase 2 is considered a tumor suppressor gene. Although the function of this gene in tumor suppression remains unclear, we speculate that in mammals, oncogenesis-associated up-regulation of ceramide synthase 2 promotes the synthesis of GSLs containing very-long-chain fatty acids and the production of antibodies that react with them, resulting in the suppression of oncogenesis. This hypothesis is also supported by the results of the present study demonstrating that the antibodies induced by immunization with CF4-C12L react with a glycoprotein specifically produced in hepatocellular carcinoma (Fig. 8). These results also indicate that CF4-C12L could serve as a seed compound in the development of vaccines against liver cancer.

In conclusion, the results of the present study revealed that artGSLs containing C12L and C182L can induce a specific adaptive immune response and the production of antibodies against the oligosaccharide as the epitope, and certain oligosaccharide structures, such as a terminal mannose, can enhance the IgG-inducing activity. As artGSLs incorporating these structural features efficiently induce the production of antibodies reactive with various oligosaccharides, they could be used to develop anti-glycoconjugate antibodies and vaccines for cancers associated with aberrant glycosylation.

## Methods

### Materials

Synthesis of artGSLs via organic chemistry was carried out by Wako Pure Chemical Industries, Ltd. (Osaka, Japan) and Tokyo Chemical Industry Co., Ltd. (Tokyo, Japan). Details of the artGSL synthesis procedure are found in Japan patent 6143240 and United States patent US10307471B2. Lipid A from *Salmonella enterica*, fetuin from fetal calf serum, and thyroglobulin from porcine thyroid gland were obtained from Sigma-Aldrich (St. Louis, MO, USA). The presence of 6′-sialyl LacNAc in fetuin was confirmed as reported previously^12^, and the core fucose epitope of thyroglobulin was confirmed using horseradish peroxidase (HRP)-linked *Lens culinaris* agglutinin (LCA, J-OIL MILLS, Tokyo, Japan). AFP-L3 and AFP-L1 were obtained from Wako Pure Chemical Industries. Synthetic αGalCer was purchased from Tokyo Chemical Industry. Glycoprotein extraction was conducted according to a previously reported method^37–39^ with slight modifications. Glycoproteins containing sLe^X^ and Le^X^ epitopes were obtained by extraction from 1 × 10^7^ HL-60 cells (provided by the RIKEN Cell Bank, Tsukuba, Japan) in 1 ml of 20 mM HEPES/0.25 M sucrose buffer (pH 7.5) supplemented with 0.1 mM phenylmethylsulfonyl fluoride and a proteinase inhibitor cocktail (Complete mini EDTA-free; Roche Diagnostics Gmbh, Mannheim, Germany) using a tight-fitting Dounce homogenizer (total of 25 strokes). After centrifugation of the homogenates at 800 *g* for 15 min at 4 °C, the supernatants were filtered using an Ultrafree-MC (0.45 μm) centrifugal device (Millipore, Billerica, MA, USA) at 13,000 *g* for 3 min at 4 °C. The filtrates were used as glycoprotein samples in subsequent experiments. The concentration of protein in each sample was determined using a Bio-Rad protein assay (Bio-Rad Laboratories, Hercules, CA, USA). sLe^X^ and Le^X^ epitopes in this sample were confirmed by ELISA using anti-sLe^X^ (KM93, Millipore) and anti-Le^X^ antibodies (MC-480, R&D Systems, Minneapolis, MN, USA), respectively.

### Immunization and preparation of serum

C3H/HeN mice (CLEA Japan, Tokyo) were used in this study. Mice were immunized with artGSLs according to a liposome immunization method^12,22^. In brief, 100 μg of artGSL was mixed with 10 μg of lipid A, 0.5 μmol of cholesterol, and 0.5 μmol of dipalmitoylphosphatidylcholine. The mixture was then dissolved in PBS and used as an immunogen. Mice were initially immunized subcutaneously and then intraperitoneally 2 weeks after the first immunization. Serum was prepared from blood collected from the tail vein 3 or 7 days after the second immunization. The Committee for Experiments Involving Animals of the National Institute of Advanced Industrial Science and Technology (AIST) approved all animal experiments, and all experiments were performed in accordance with relevant guidelines and regulations.

### ELISAs

ELISAs were performed as described previously^12^. In brief, 500 ng of artGSL, 500 ng of AFP-L1 and -L3, and 1 μg of other glycoproteins was applied to the wells of a 96-well microtiter plate and were incubated overnight. After washing twice with PBS, blocking buffer (1% bovine serum albumin in PBS) was added to each well and incubated for 15 min at room temperature, followed by the addition of diluted serum (1:50 for IgG, 1:100 for IgM and total immunoglobulin). After incubation for 3 h at room temperature, the wells were washed with 0.1% Tween 20 in PBS, and then an HRP-linked secondary antibody (anti-IgM or anti-IgG or anti-Ig) was added. Antibody binding was detected using an HRP substrate (1-Step Ultra TMB-ELISA Substrate; Thermo Fisher Scientific, Waltham, MA, USA) and measurement of absorbance at 450 nm. The immobilization efficiency of 6′-sialyl LacNAc-C12L derivatives on the microtiter plate were shown in Fig. S5. The result indicates that C24 fatty acid structure is important for the efficiency but the sphingosine structures are hardly influenced to the efficiency.

### Serum cytokine analysis

Liposomes composed of 0.5 μmol of cholesterol, 0.5 μmol of dipalmitoylphosphatidylcholine, and 10 μg of αGalCer or 100 μg of artGSL were dissolved in PBS and injected intraperitoneally into C3H/HeN mice. Blood was collected from the tail vein of mice at specific time points (see Fig. 6), and then serum samples were prepared. Levels of IL-4 and INF-γ in the serum were determined using mouse IL-4 and INF-γ ELISA kits (Thermo Fisher Scientific).

### Characterization of serum immunoglobulin isotypes

Serum was diluted 500-fold with 1% bovine serum albumin in PBS, and the isotype of antibodies present in the serum was determined using a mouse monoclonal antibody isotyping kit (Roche Diagnostics GmbH) according to the manufacturer’s instructions.

### Statistical analysis

After determination of variance using the F-test, the statistical significance of differences in data was evaluated using the two-tailed Student’s *t*-test, with statistical significance defined as follows: *P < 0.05, **P < 0.01, and ***P < 0.001. Samples with similar average values between groups were excluded from this test.

## Supplementary information

Supplementary Figures

## Data Availability

The datasets generated during and/or analyzed during the current study are available from the corresponding author on reasonable request.
