# Supplementary material for: Induction of specific adaptive immune responses by immunization with newly designed artificial glycosphingolipids
Source: Sci Rep. 2019 Dec 11;9:18803. doi: 10.1038/s41598-019-55088-9 (PMC6906409; doi:10.1038/s41598-019-55088-9)
Supplement: Supplementary file 1 — Supplementary Figures [file 41598_2019_55088_MOESM1_ESM.pdf]

Induction of specific adaptive immune responses by immunization with newly designed artificial glycosphingolipids

Tetsuya Okuda[1*], Kayoko Shimizu[2], Satoshi Hasaba[2], Mutsuhiro Date[2]

1. Bio-Design Research Group, Bioproduction Research Institute, National Institute of Advanced Industrial Science and Technology (AIST), Central 6, 1-1-1 Higashi, Tsukuba, Japan

2. Diagnostics Research Laboratories, Diagnostics Technical Service & Research Operations, Diagnostics Division, FUJIFILM Wako Pure Chemical Corporation, 6-1 Takada, Amagasaki, Hyogo, Japan.

Supplementary Information

Supplemental Figure S1
Supplemental Figure S2
Supplemental Figure S3
Supplemental Figure S4
Supplemental Figure S5

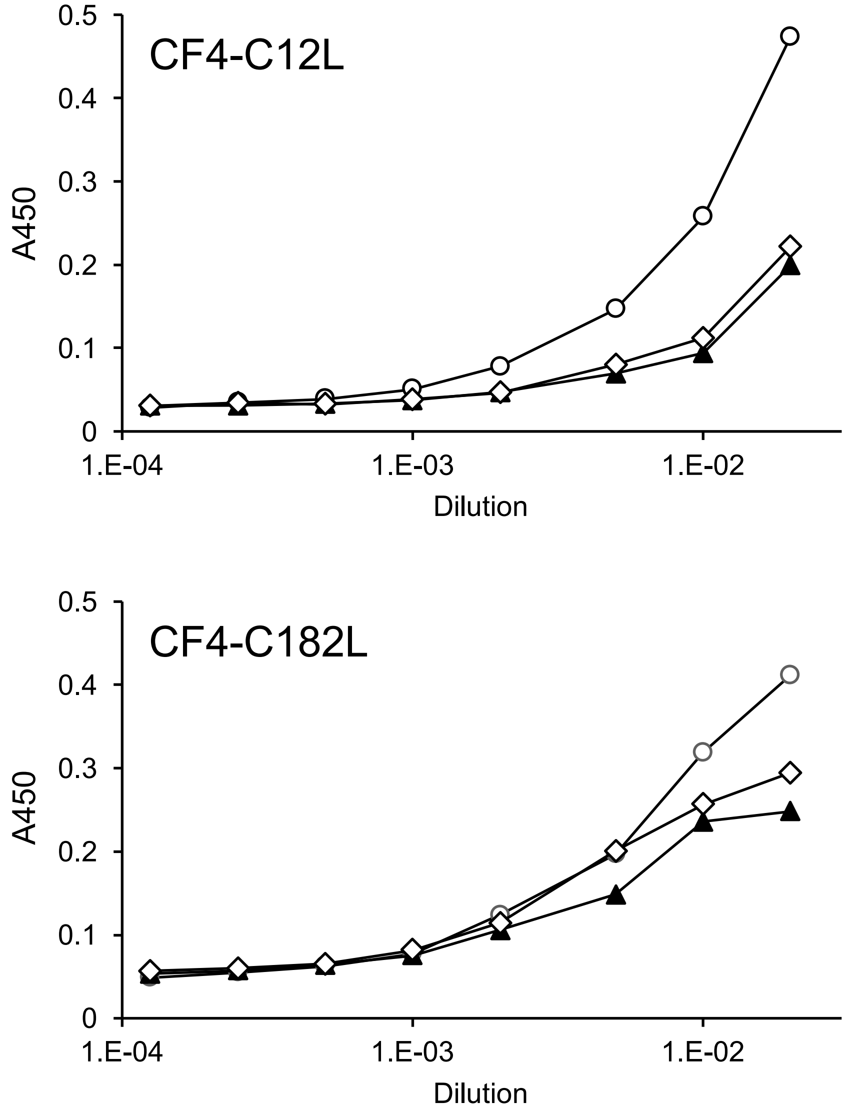

Figure S1. Anti-immunogen IgG titers in diluted sera from CF4-C12L or CF4-C182L immunized mice. The D7 sera of mice with high antibody titer in Fig. 5 were analyzed (n=3). The *open circles*, *open squares*, *and closed triangle* indicate the serum from individual mouse. Serum IgG titers against each immunogen were determined by ELISA as described in the *Methods*.

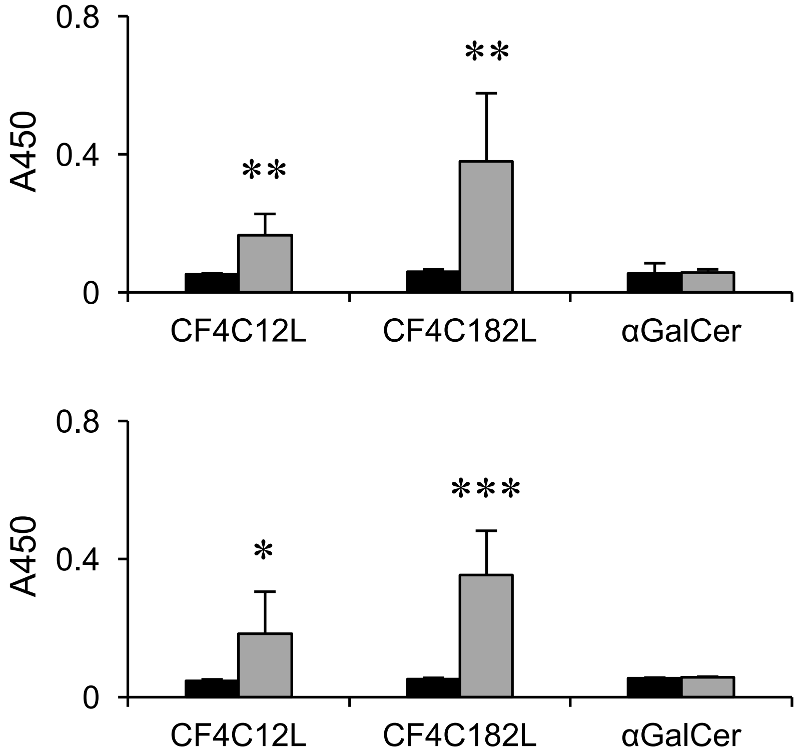

Figure S2. Titers of immunogen-specific serum antibodies in αGalCer-immunized mice. Mice were immunized with αGalCer, and serum was prepared 7 days (*gray bars*) after the final immunization. *Closed bars*, serum from non-treated mice. Titers of serum IgM (*upper panel*) and IgG (*lower panel*) against αGalCer were determined by ELISA. Titers of immunogen-specific serum antibodies in CF4-C12L– and CF4-C182L–immunized mice are shown for reference. Error bars: mean ± S.D. (n = 4-7). **P* < 0.05, ***P* < 0.01, ***P* < 0.001 BG vs. D7.

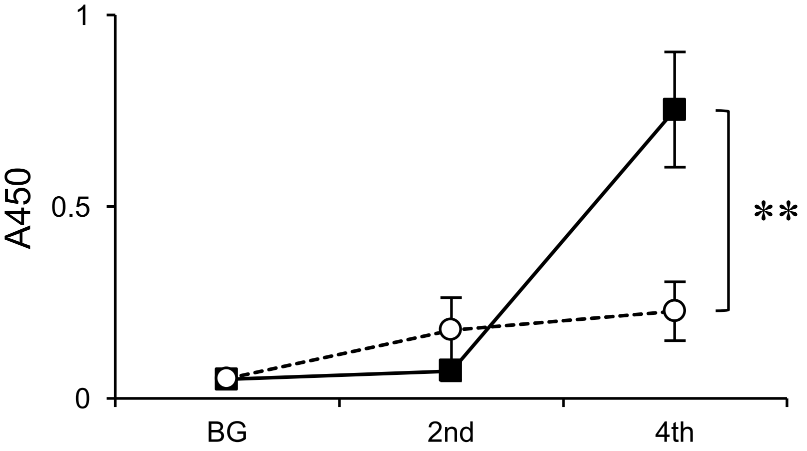

Figure S3. Serum antibody titers in mice repetitively immunized with CF4 conjugates. Serum of mice immunized more than 4 times with CF4-C12L (*closed squares with solid line*) or CF4-C182L (*open circles with dotted line*) was prepared 3 days after the final immunization (4th). Titers of serum IgG against each immunogen were determined by ELISA. BG, serum from non-treated mice; 2nd, serum from second booster immunization. Error bars: mean ± S.D. ($n = 3$-4). $^{**}P < 0.01$ anti-CF4-C12L vs. anti-CF4-C182L.

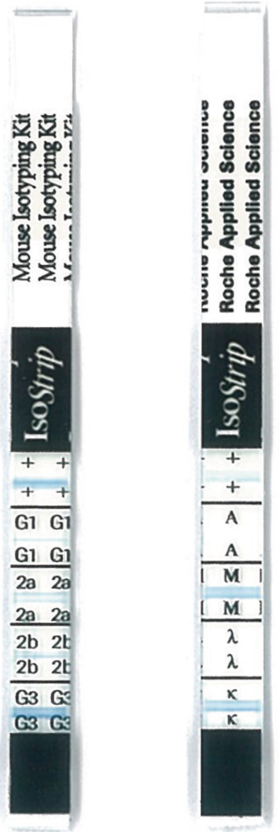

Figure S4. Serum immunoglobulin isotypes in CF4-C12L–immunized mice. A mouse (No. 3 in Fig. 8) was repetitively immunized with CF4-C12L, and serum was prepared 3 days after the final immunization. Figure shows serum immunoglobulin isotypes determined using a mouse antibody isotyping kit.

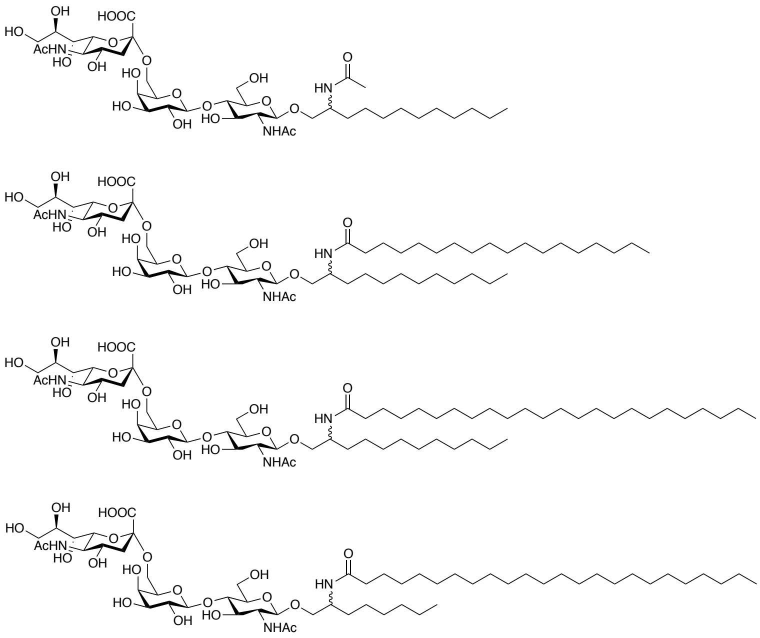

C12-2

C12-18

C12-24 (C12L)

C8-24

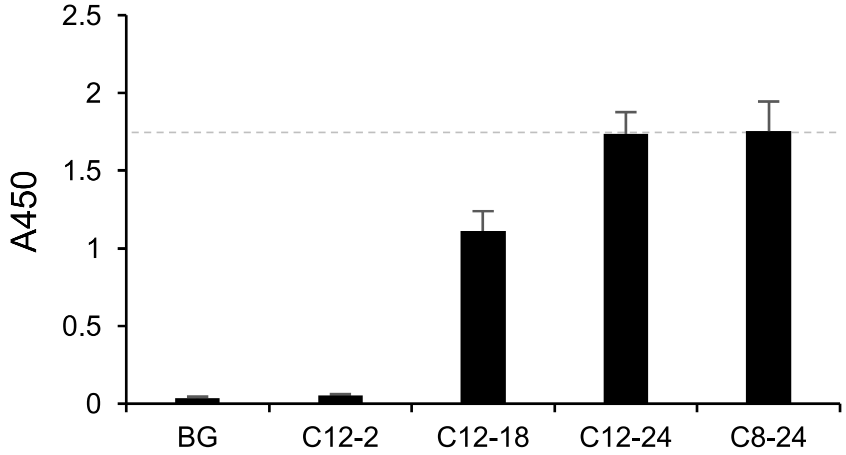

Figure S5. Immobilization efficiency of artificial glycolipid on microtiter plate. A 96-well microtiter plate was coated with 500 ng of several derivatives of ceramide portion of the 6'-sialyl LacNAc-C12L. Immobilized efficiency was determined by binding of an anti-6'-sialyl LacNAc antibody FR9 as described in the *Methods*. BG: absorbance of uncoated wells. Error bars: mean ± S.D. (n = 3).
